# The Bergen Facebook Addiction Scale: its psychometric properties and invariance among women with eating disorders

**DOI:** 10.1186/s12905-022-01677-2

**Published:** 2022-03-31

**Authors:** Amira Mohammed Ali, Amin Omar Hendawy, Eman Sameh Abd Elhay, Esraa Mohammed Ali, Abdulmajeed A. Alkhamees, Hiroshi Kunugi, Nagia I. Hassan

**Affiliations:** 1 Department of Psychiatric Nursing and Mental Health, Faculty of Nursing, Alexandria University, Smouha, Alexandria, 21527 Egypt; 2 Department of Biological Production, Tokyo University of Agriculture and Technology, Fuchu, Tokyo, 183-8538 Japan; 3 Department of Animal and Poultry Production, Faculty of Agriculture, Damanhour University, Damanhour, 22516 Egypt; 4 Psychiatric and Mental Health Nursing Department, Faculty of Nursing, Mansoura University, Mansoura, 30016 Egypt; 5 Department of Basic and Educational Sciences, Faculty of Education for Early Childhood, Alexandria University, Mostafa Kamel, Alexandria, 21646 Egypt; 6 Department of Medicine, Unayzah College of Medicine and Medical Sciences, Qassim University, Unayzah, 52571 Al Qassim Saudi Arabia; 7 Department of Psychiatry, Teikyo University School of Medicine, 2-11-1 Kaga, Itabashi-ku, Tokyo, 173-8605 Japan; 8 Department of Mental Disorder Research, National Institute of Neuroscience, National Center of Neurology and Psychiatry, 4-1-1, Ogawahigashi, Kodaira, Tokyo, 187-8502 Japan; 9 Department of Psychiatric Nursing and Mental Health, Faculty of Nursing, Damanhour University, Damanhour, 22511 Egypt

**Keywords:** Bergen Facebook Addiction Scale/Facebook dependence/problematic Facebook use/social networking addiction, Eating disorders/anorexia nervosa/bulimia nervosa/binge eating, Factorial structure/structural validity/validation/invariance/psychometric properties, Six-item Internet Addiction Test/short version of the Internet Addiction Test, Women

## Abstract

**Objective:**

Facebook addiction is increasing, giving rise to limited real-life social networks, loneliness, poor work and academic performance, psychopathology, and low well-being. Facebook entails numerous factors that increase the risk for disordered eating attitudes and behaviors (e.g., use time and Facebook activities such as social grooming and photo sharing). This study aimed to evaluate the psychometric properties of the Bergen Facebook Addiction Scale (BFAS) among patients with eating disorders (EDs) given lack of validation of Facebook addiction measures in this population.

**Methods:**

A cross-sectional study involving 123 inpatient and outpatient women with EDs (Mean age = 27.3, SD = 10.6, range = 14–59 years) used confirmatory factor analysis (CFA), multigroup CFA, structural equation modeling (SEM), Spearman’s rho Spearman’s analysis, McDonald's Omega (ω), Cronbach’s alpha (α), and item-total correlations to examine the structure, invariance, criterion validity, reliability, and discriminant validity of the BFAS.

**Results:**

Correlating the residuals of items 2, 3, and 5 resulted in an excellent fit of a one-factor structure of the BFAS (*χ*^2^(7) = 8.515, *p* = .289, CFI = .998, TLI = .996, RMSEA = .042, SRMR = .0099). The BFAS was invariant at the configural, metric, and scalar levels across groups of EDs, age, education, and marital status. High values of ω and α (.96) as well as item-total correlations (.851–.929) indicated excellent reliability and high discrimination index of the BFAS.  Criterion validity is noted by strong positive correlation with the Six-item Internet Addiction Test (S-IAT, r = .88) and SEM using the S-IAT to predict the BFAS (χ2(49) = 103.701, p = .001, CFI = .975, TLI = .966, RMSEA = .096, SRMR = .0317)..

**Conclusion:**

The BFAS is a reliable unidimensional measure. Its high discrimination index and invariance across different groups make it useful for detecting Facebook addiction among patients with ED.

**Supplementary Information:**

The online version contains supplementary material available at 10.1186/s12905-022-01677-2.

## Introduction

Eating disorders (EDs) are psychiatric conditions characterized by dysfunctional eating habits, which are associated with adverse mental and physical effects. EDs primarily strike adolescent and emerging adult women while men represent only 10% of all cases [1, 2]. The prevalence of EDs in the United States (US), Asia, and Europe is 4.6%, 3.5%, and 2.2%, respectively [3]. EDs account for the highest mortality among all psychiatric disorders [3, 4]. In addition, the total economic cost of EDs is quite high—around $400 billion in the US during fiscal year 2018–2019, including the cost of reduced wellbeing as indicated by disability-adjusted life years [3].

Up to 89% of adolescents diagnosed with EDs experience another debilitating psychiatric comorbidity [5]. Psychiatric comorbidities in EDs are associated with a 48% reduction in yearly earnings [1]. Major depressive disorder and suicide risk express the highest cooccurrences (52.4% and 40.0%, respectively) [5]. Post-traumatic stress disorder is reported in up to 17.5%, 20.7%, and 21.5% of patients diagnosed with anorexia nervosa (AN), bulimia nervosa (BN), and binge ED (BED) [6]. Comorbidity in EDs may have increased given physical inactivity and the accelerated prevalence of depressive, anxiety, and traumatic symptoms in the general population over the course of the COVID-19 outbreak [7–9]. Alcohol and/or illicit substance abuse prevails in 50% of patients with EDs compared with 9% of the general population [10]. The consumption of alcohol and illicit substances among ED patients has considerably increased since the outbreak of the COVID-19 pandemic in response to increased anxiety levels, dieting behaviors, as well as concerns about relapse and unavailability of foods consistent with meal plan [11]. Conditions comorbid with EDs display shared neurobiological mechanisms, personality features, as well as environmental and genetic factors with EDs [1, 2, 10]. For example, EDs and addictive disorders are primarily characterized by increased impulsivity, loss of control, emotional negativity, and risk for self-destructive behaviors, which all prolong the course of the disorder and lower quality of life [2, 10].

The symptoms (e.g., excessive dieting despite being underweight) and the progress of EDs may be challenging for patients’ family members who may tend to react with high expressed emotion (e.g., criticism, hostility, overprotection), resulting in inadvertent perpetuation of the problem [12]. An investigation of the effect of acute psychosocial stress on affective states and ED-related attitudes in people with AN and BN revealed that psychosocial stress causes a remarkable increase in anxiety, which is related to exacerbations in eating symptomatology and accelerated levels of interpersonal sensitivity [13]. Self-reported social isolation is common among individuals with EDs, and it is likely to propagate obsessions with unhealthy eating behaviors [14]. Indeed, perceived isolation and loneliness are major contributing factors to relapse among patients receiving treatment for EDs [14, 15].

Loneliness in EDs can be better explained within the context of the Cognitive-Interpersonal Maintenance Model, which proposes that cognitive, socio-emotional and interpersonal deficiencies across EDs interact together to cause and maintain eating psychopathology [12, 16]. This theory gets support from imaging and clinical studies. A systematic review reports dysfunction in six areas of social cognition: theory of mind, social perception, social knowledge, attributional bias, emotion perception, and emotion processing, primarily in patients with AN [16]. Impairments in set shifting and global integration during the ill state of the disorder contribute to weak central coherence [12]. Vivid alterations in socio-emotional processing in patients with AN include impaired signaling of, interpretation and regulation of emotions as well as an automatic bias in attention towards critical and domineering faces and away from compassionate faces [12]. Patients with BN and BED express some deficits in social cognition, but research reports are inconsistent [16]. In this respect, women with BN undergoing experimental exposure to social interactions with negative reciprocity are reported to express more negative behavioral responses and stronger neural activations in the cortical and subcortical brain regions than healthy women. On the other hand, exposure to positive reciprocity interactions was not associated with any behavioral or neural differences, denoting amplification of negative self-relevant social interactions in BN [17]. Similar alterations (e.g., subcortical activations involving the amygdala and dorsal striatum) are noticed in the acute phase in those patients [17], denoting that negative social interactions represent manifest triggers of ED pathologies.

People with EDs demonstrate a tendency for problematic internet use, which is probably more directed toward the use of social media as a way to cope with negative emotions and loneliness—lonely individuals addicted to gaming on social networking sites (SNSs) seek the reward of “inclusion in a social group” [18, 19]. SNSs are virtual communities where users (1) construct a public or semipublic profile within a bounded system, (2) articulate and interact with a list of other users (including real-life friends) based on shared interests, and (3) view and traverse their own connections and those of others within the system [19]. Facebook is the most popular form of SNSs [20, 21]. Individuals are thought to use SNSs to meet their needs for belonging and affiliating with others and for self-presentation. However, youth and individuals with certain personality traits may quickly develop excessive use and lack of control [21]. Excessive and problematic use of SNSs is referred to as social networking addiction—people get addicted to cyber-relationships and the rewards gained from interacting with people within their friendship networks [22].

According to the “biopsychosocial framework” for the etiology of addictions and the “syndrome model of addiction” shifting from normal to social networking misuse in social network addiction develops when social networking becomes an important or even exclusive means to cope with stress, loneliness and depression, which are common features in individuals with substance addiction [21]. In support of these theories, people who are lonely, single [23], high on social anxiety [24], not in love relation, failing love relation [25], or lacking social support [26] express more internet and Facebook dependence than counterparts who are less lonely, married, in love relations or have adequate social support. Incidentally, longitudinal investigations emphasize that loneliness increases over time as a consequence of prolonged Facebook/internet use, and online interactions with family and friends are unable to counteract loneliness feelings [27]. Longitudinal data also show that prolonged Facebook use is associated with a significant increase in the number of people reaching the critical cut-off scores of Facebook addiction [28].

Same as in other addictive disorders, social media addiction results in numerous negative consequences, including interpersonal conflicts and relationship problems, shrinkage in real-life social networks, deterioration of academic performance, reduction in general well-being [29, 30], and increased prevalence of depression (OR 5.11, 95% CI 1.42–18.34) in analysis adjusted for other characteristics [31]. An interesting investigation of a wide range of desires among Facebook and Twitter users in Germany revealed that desires for social media use are more dominant than desires for rest and sleeping, which were most prominent among the participants. They are also more experienced than desires for smoking and alcohol drinking, which are addictive in nature. Thus, problematic use of social media may be more detrimental than addictive substances given the wide availability and cheapness of social media [32].

Symptoms of dysfunctional eating are more likely to be internet dependent while internet addiction is a confirmed predictor of all subtypes of EDs among university students [33, 34]. Indeed, meta-analytic data confirm causal claims associating SNSs/Facebook use and disordered eating [35]. Facebook implicates numerous factors that increase the risk for eating pathology such as media and peers [34, 36, 37]. These factors operate via a mechanism that involves potentiation of emotional negativity, which furthers the progression to disordered eating in persons addicted to Facebook/internet [34]. In particular, time spent online and frequent Facebook use are associated with increased state anxiety, depressive symptoms, concerns about weight/shape/eating, as well as disordered eating attitudes and behaviors [36, 38]. Longitudinal studies indicate that specific Facebook activities such as social grooming (e.g., viewing and commenting on peers’ profiles), seeking and receiving negative feedback via SNSs [39, 40], and photo-based Facebook activities rather than use time contribute to increased body dissatisfaction, derive for thinness, and disordered eating [36, 37, 41]. Appearance comparison mediates this relationship [39]. Given the role of Facebook addiction in the initiation and maintenance of eating pathologies and their related derives (e.g., emotional negativity, body dissatisfaction, and loneliness), calibrated measures are intensely needed for accurate detection of Facebook addiction in individuals diagnosed with or prone to EDs.

The Bergen Facebook Addiction Scale (BFAS) was developed by Andreassen and colleagues to measure problematic Facebook use as a specific type of internet addiction. The authors selected six items from a pool of 18 items evaluating different addiction components, with each item representing one component (e.g., salience, conflict, tolerance, etc.) [30]. Suggested cut-off points for diagnosing problematic Facebook use are a total score of 12 or 18 based on liberal and conservative approaches [30, 42]. A single change in the BFAS involving the replacement of the word “Facebook” with “SNSs” resulted in a new scale known as the Bergen Social Media Addiction Scale (BSMAS). SNSs were defined as Facebook, Twitter, Instagram and the like [20]. Investigations involving the BFAS and BSMAS among users of Facebook and at least one more SNS show that both measures can be used synonymously [20].

Several studies from different countries have examined the construct validity of the BFAS, primarily among adolescents, university students, and emerging adults [20, 24, 42–46]. All studies support the one-factor structure, which was originally described by Andreassen [30]. However, the one-factor model exhibited misfit in some studies, which was remedied by allowing some residuals to correlate [20, 24, 42]. Suboptimal loadings for few items are also reported [47]. Investigations of invariance of the BFAS across different groups are few, and the results are inconsistent denoting invariance [43] or minimal non-invariance across gender groups [42] and varying levels of non-invariance across groups of age [42, 43] and academic achievement [43].

While Facebook addiction increases the risk for EDs by inducing mood dysfunctions [36], it is also highly prevalent in affective disorders and anxiety disorders (up to 28.6% and 0.9%) [37], which frequently cooccur in EDs [5, 6]. Longitudinal studies indicate that Facebook addiction in these conditions reduces positive mental health, resulting in increases in depressive symptoms and insomnia—common indicators of poor recovery [48]. Facebook withdrawal symptoms (e.g., restlessness, depression, anxiety) occur in up to 86.6% of those patients [37]. Therefore, the negative consequences of Facebook addiction in EDs (e.g., delayed recovery) can be further exacerbated by the presence of other psychiatric comorbidities. Nonetheless, the psychometric properties of the BFAS have not been evaluated among patients with EDs or any other physical or mental disorder. The current study aimed to fill the existing gap by evaluating the structure of the BFAS and its invariance among patients with EDs. We hypothesized that the BFAS would (1) exhibit a unidimensional structure, (2) demonstrate non-invariance across groups of EDs, age, education, and marital status, and (3) positively correlate with internet addiction among women with EDs.

## Material and methods

### Study participants and procedure

This study is based on a sample of 124 women recruited consecutively from the inpatient and outpatient units of the General University Hospital of Ciudad Real in Spain during the period between February and November 2018. Inclusion criteria were (1) having a diagnosis of EDs, (2) being aged above 12 years, (3) having no physical or mental disabilities, and (4) accepting to sign an informed consent. The legal guardians of participants younger than 18 years signed informed consent [49]. All experimental protocols were approved by the Ethics and Clinical Research Committee of Ciudad Real (Spain) (ref. 2017C/123) [49]. However, no ethical approval was attained for the present study since the analysis is based on a publicly accessible dataset [50].

### Data collection tools

Data collection tools have been previously described elsewhere [49]. In brief, the sociodemographic and clinical characteristics (e.g., history of hospitalization, smoking status) were collected by a set of questions.

### The Bergen Facebook addiction Scale

The Bergen Facebook addiction Scale (BFAS) was used to evaluate problematic use of Facebook. The BFAS is a six-item measure, inquiring about specific components of addiction related to Facebook e.g., spending a lot of time thinking about or planning to use, having an urge to use, feeling restless when prohibited from use, having negative effects on job/study because of use, and trying to cut down use. Responses to the BFAS are provided on a 5-point Likert scale that ranges between very rarely = 1 to very often = 5. Problematic use is indicated by scores higher than 12 or 18 [30].

### The Six-item Internet Addiction Test

The Spanish version of Young’s 20-item Internet Addiction Test (IAT) [51] has been refined based on Griffiths’ addiction component model [52] into the Six-item Internet Addiction Test (S-IAT) [53]. Each item depicts a specific component, including salience (feel preoccupied with the internet), mood modification (block disturbing thoughts about your life), conflict (your work suffers), tolerance (find yourself saying “just a few more minutes” when online), withdrawal (feel depressed, moody, or nervous when you are offline), and relapse (try to cut down the amount of time you spend online and fail). Responses are rated on a five-point Likert scale (0 = not applicable, 1 = never, 2 = rarely, 3 = sometimes, 4 = often, and 5 = very often). The total score of S-IAT ranges between 0 and 30. Scores below 12 indicate normal use, scores between 12 and 18 indicate problematic use, and higher scores indicate internet addiction. Because the psychometrics of the S-IAT are more robust than the IAT [53], it was used in the analysis to evaluate the criterion validity of the BFAS.

### Statistical analysis

One observation was removed from the sample for having incomplete data on one of the key variables. Quantitative variables with normal and non-normal distribution were described using mean and standard deviation or median and interquartile range (IQR: 25–75%). Categorical variables were described using number and percentage.

Confirmatory factor analysis (CFA), involving maximum likelihood with bootstrapping based on 2000 random replications, was used to check data-fit to the one-factor structure of the BFAS in the whole sample (*N* = 123) and two main groups of EDs (women with AN constituted group 1 (*n* = 59) while women with other EDs subtypes constituted group 2 (*n* = 64)). This classification is based on significant between group differences in body mass index, t(73.0) =  − 6.77, *p* = .001 [2]. To judge data-model-fit as good or acceptable, in order, we used Comparative Fit Index (CFI) and Tucker–Lewis Index (TLI) equal to or above .95 and .90, respectively. In addition, values of the root mean square error of approximation (RMSEA) and the standardized root-mean-square residual (SRMR) should be less than .06 and .08, respectively [9, 54]. Suggestions indicated by modification indices were considered for improving model fit.

Invariance of the crude structure of the BFAS across groups of EDs, age, education, and marital status (see Table 1 for groups) was examined via multigroup CFA. To affirm measurement invariance of the BFAS across groups, we examined data fit to four models. Configural invariance is judged based on unconstrained model indicating general fit of the model across groups. Metric invariance is based on constraining factor loadings to be equal between groups and examining the difference between the unconstrained and constrained model. Strong/scalar invariance is based on constraining the intercepts of the items to be the equal between groups. Strict invariance is based on constraining residuals to be equal between groups [55, 56]. Although a significant *χ*^2^ may reflect changes in model fit across groups, it is largely dependent on sample size, which may disqualify proper-fitting models that express minor misspecifications [57–59]. Therefore, deteriorations in model fit following imposing constraints between groups can be better reflected by changes in CFI and RMSEA, which are less dependent on sample size. For invariant models, ΔCFI and ΔRMSEA should not exceed 0.02 and 0.015, respectively [60, 61].

**Table 1:** Sociodemographic characteristics of the participants in the samples

Participants’ characteristics	*N* = 123
	No (%)
Age in years	
20 years and less	42 (34.1)
Above 20 years	81 (65.9)
Subtypes of eating disorders	
Anorexia nervosa	59 (48.0%)
Bulimia nervosa	35 (28.5%)
Binge eating disorders	11 (8.9%)
Eating disorders not otherwise specified	18 (14.6%)
Marital status◆	
Single	100 (81.3)
Married	22 (17.9)
Education	
School education	83 (67.5)
University	40 (32.5)
BMI mean (SD)	22.2 (8.4)
Facebook addiction	
Normal use (BFAS < 12)	67 (54.5)
Problematic use (BFAS = 12–18)	22 (17.9)
Facebook addition (BFAS > 18)	34 (27.6)
Internet addiction	
Normal use (S-IAT < 12)	96 (87.0)
Problematic use (S-IAT = 12–18)	11 (9.0)
Facebook addition (S-IAT > 18)	16 (13.0)

◆: one observation is missing, *BMI* body mass index, *SD* standard deviation, *BFAS* Bergen Facebook Addiction Scale, *S-IAT* Six-item Internet Addiction Scale

We examined the reliability of the BFAS using McDonald's Omega coefficient (ω), as well as Cronbach's alpha coefficient (α) and item-total correlations. Criterion validity of the BFAS was examined by structural equation modeling (SEM) involving maximum likelihood with bootstrapping based on 2000 random samples using the S-IAT to predict the BFAS. Spearman’s rho correlation between the scores of the S-IAT and the BFAS was also conducted. SPSS version 22 and Amos version 24 were used for the analysis, and significance was considered at a probability of .05, two-tailed.

## Results

### Participants’ characteristics

This cross-sectional study comprised a convenient sample of 123 women diagnosed with various subtypes of EDs (Mean age = 27.3, SD = 10.6, range = 14–59 years). The median (IQR) of Facebook addiction and internet addiction was 11 (7–19) and 4 (1–11), respectively. Other sociodemographic and clinical characteristics of the sample are described in Table 1.

### Confirmatory factor analysis

**Fig. 1:**
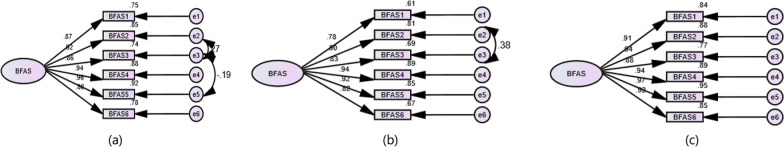
Confirmatory factor structures of the Bergen Facebook Addiction Scale expressing the best fit among all women with eating disorders (**a**), women with anorexia nervosa (**b**), and women with other subtypes of eating disorders (**c**)

Examination of the BFAS by CFA revealed excellent fit of the unidimensional structure of the BFAS in the group of other EDs as indicated by values of all fit indices. *χ*^2^ was significant and the values of RMSEA were considerably high in the whole sample and in women with AN, indicating unacceptable fit. Modification indices revealed improvement in model fit by allowing error terms to correlate (item 3 with both item 2 and item 5 in the whole sample and item 2 with item 3 in women with AN, Fig. 1), Model 2. In both models, all item loadings were significantly high on the extracted factor (*p* < .001), Table 2.

**Table 2:** Goodness-of-fit indices for models of the Bergen Facebook Addiction Scale examined in confirmatory factor analysis

Models	Samples	*χ*^2^	*p*	*df*	Factor loadings	CFI	TLI	RMSEA	RMSEA 95% CI	SRMR
Model 1 (C)	*N* = 123	20.28	.016	9	.86 to .95	.987	.979	**.101**	.041 to .161	.0147
	*n* = 59	14.67	.100	9	.79 to .93	.983	.972	**.104**	.000 to .197	.0249
	*n* = 64	10.30	.281	9	.88 to .97	.996	.994	.058	.000 to .160	.0128
Model 2 (E)	*N* = 123	8.52	.289	7	.86 to .96	.998	.996	.042	.000 to .124	.0099
	*n* = 59	8.20	.414	8	.78 to .94	.999	.999	.021	.000 to .156	.0216

*χ*^2^, chi-square; df, degrees of freedom; CFI, comparative fit index; TLI, Tucker–Lewis index; RMSEA, root mean square error of approximation; CI, confidence interval; SRMR, standardized root mean residual; C, crude models; E, models with correlated error terms, values in boldface indicate misfit

### Invariance of the BFAS across different groups

Model 1 was used to test invariance across different groups. This is because when model 2 was used, considerable non-invariance at the scalar level was revealed across EDs groups (Additional files 1 and 2). As shown in Table 3, the BFAS was invariant at the configural, metric, scalar, and strict levels across groups of education. *χ*^2^ was significant in three models indicating scalar non-invariance across EDs groups and strict non-invariance across groups of age and marital status. However, the values of ΔCFI and ΔRMSEA were below the levels that signal non-invariance, except for marital status (ΔCFI = .030 and ΔRMSEA = − .019), denoting real non-invariance of the BFAS at the strict level between single and married participants.

**Table 3:** Invariance of the factor structures of the Bergen Facebook Addiction Scale across different groups

Groups	Invariance levels	*χ*^2^	df	*p*	Δ*χ*^2^	Δdf	*p*(Δ*χ*^2^)	CFI	ΔCFI	TLI	ΔTLI	RMSEA	ΔRMSEA	SRMR
Eating disorders	Configural	25.60	18	.109				.991		.986		.059		.0249
	Metric	30.98	23	.123	5.38	5	. 371	.991	.000	.988	− .002	.054	.005	.0313
	Strong	35.72	24	.058	4.74	1	.029	.987	.004	.983	.005	.064	− .010	.0616
	Strict	41.58	30	.078	5.86	6	.439	.987	.000	.987	− .004	.056	.008	.0706
Age	Configural	34.08	18	.012				.982		.970		.086		.0280
	Metric	44.07	23	.005	9.98	5	.076	.976	.006	.969	.001	.087	− .001	.0388
	Strong	44.08	24	.007	0.01	1	.914	.977	− .001	.971	− .002	.083	.004	.0397
	Strict	61.60	30	.001	17.52	6	.008	.964	.013	.964	.007	.093	− .010	.0621
Education	Configural	38.63	18	.003				.977		.962		.097		.0180
	Metric	42.38	23	.008	3.74	5	.587	.979	− .002	.972	− .010	.083	.014	.0184
	Strong	42.45	24	.011	0.07	1	.788	.980	− .001	.975	− .003	.080	.003	.0192
	Strict	53.31	30	.005	10.66	6	.093	.974	.006	.974	.001	.080	.000	.0196
Marital status	Configural	49.77	18	.001				.965		.942		.121		.0274
	Metric	62.37	23	.001	12.60	5	.027	.957	.008	.943	− .001	.119	.003	.0427
	Strong	63.16	24	.001	34.14	7	.373	.957	.000	.946	− .003	.117	.002	.0377
	Strict	**96.51**	**30**	**.001**	33.35	6	**.001**	.927	**.030**	.927	**.019**	.136	− **.019**	.0607

*χ*^2^, chi-square; df, degrees of freedom; CFI, comparative fit index; TLI, Tucker–Lewis index; RMSEA, root mean square error of approximation; CI, confidence interval; SRMR, standardized root mean residual, values in boldface indicate significant non-invariance, values in boldface indicate non-invariance

Examinations of significant differences across groups using independent sample t-test revealed no differences in the BFAS between women with AN and women with EDs (M ± SD = 12.6 ± 6.2 vs 14.2 ± 8.2, t(116.7) = − 1.202, *p* = .232). However, there were between group differences in the BFAS scores across age groups—they were significantly higher among younger compared with older patients (M ± SD = 16.3 ± 7.0 vs 12.0 ± 7.2, t(85.2) = 3.162, *p* = .002). Likewise, single patients scored higher on the BFAS than married participants (M ± SD = 14.4 ± 7.3 vs 9.4 ± 6.3, t(34.6) = − 3.247, *p* = .003).

### Criterion validity

Criterion validity of the BFAS was supported by SEM—the S-IAT predicted the BFAS in the whole sample, women with AN and women with EDs (Table 4). Correlations between the S-IAT and the BFAS in the samples are consistent with  the results of SEM (Table 4), indicating adequate convergent validity of the BFAS.

**Table 4:** Goodness-of-fit indices for models using the six-item Internet Addiction Test (S-IAT) to predict Facebook addiction on the Bergen Facebook Addiction Scale (BFAS), correlations between the S-IAT and the BFAS, and reliability of the BFAS in the samples

Samples	*χ*^2^	*p*	*df*	CFI	TLI	RMSEA	RMSEA 95% CI	SRMR	Variance %	Correlation with S-IAT	Cronbach’sα	McDonald’sω	McDonald’s ω 95% CI
*N* = 123	103.70	.001	49	.975	.966	.096	.070 to .121	.0317	79	.88**	.96	.96	.95 to .97
*n* = 59	77.48	.008	50	.965	.953	.097	.051 to .138	.0600	66	.82**	.95	.94	.91 to .96
*n* = 64	100.80	.001	51	.962	.951	.125	.088 to .160	.0268	85	.88**	.97	.97	.96 to .98

*χ*^2^, chi-square; df, degrees of freedom; CFI, comparative fit index; TLI, Tucker–Lewis index; RMSEA, root mean square error of approximation; CI, confidence interval; SRMR, standardized root mean residual; S-IAT, six-item Internet Addiction Test, α, alpha, ω, omega, **p < .01

### Internal consistency

The BFAS expressed excellent reliability in the samples (Table 4), item-total correlations (range = .95 to .96) shown in Table 5 indicate adequate discrimination ability of items of the BFAS i.e., the scale can differentiate individuals with problematic Facebook use from those with normal use.

**Table 5:** Item analysis of the Bergen Facebook Addiction Scale in the sample (*N* = 123)

	Items	Mean	SD	Scale mean if item deleted	Scale variance if item deleted	Item-total correlation	Cronbach'sαif Item deleted
1	Spent a lot of time thinking about Facebook or planned use of Facebook	2.0	1.1	11.55	41.17	.85	.96
2	Felt an urge to use Facebook more and more	2.4	1.4	11.31	37.00	**.92**	.95
3	Used Facebook in order to forget about personal problems	2.8	1.3	11.33	36.36	**.93**	.95
4	Tried to cut down on the use of Facebook without success	2.2	1.4	11.40	36.96	.87	.96
5	Become restless or troubled if you have been prohibited from using Facebook	2.1	1.4	11.09	36.74	**.92**	.95
6	Used Facebook so much that it has a negative impact on your job/studies	2.1	1.4	10.72	38.07	.85	.96

Values in boldface show that items with correlated error terms express higher item-total correlations, denoting their relevance to the latent structure

## Discussion

Problematic Facebook use is on the rise [20]. It has a causal effect on eating disorder symptoms and attitudes [35–37, 40, 41]. Comorbid dependence on Facebook/internet increases emotional negativity (e.g., depression and state anxiety) in individuals prone to disordered eating [34, 36]. It also may increase distress and worsen psychopathology in other psychiatric conditions, which are frequently comorbid with EDs such as affective disorders [37, 48]. Therefore, the current study examined the psychometric properties of the BFAS among women with EDs through a set of robust techniques. The study enriches existing knowledge by revealing that the BFAS is a rigorous measurement tool, which may facilitate the detection of problematic Facebook use among individuals with EDs.

The BFAS expressed good fit for a one-factor structure in patients with EDs (Table 2). The psychometrics of the BFAS have not been described in any patient population. However, our findings are congruent with previous studies, which recruited adolescents, students, and healthy adults [20, 24, 43, 44]. As shown in Fig. 1, the fit of the one-factor model in the whole sample and in patients with AN was improved by correlating the residual of item 3 “used Facebook in order to forget about personal problems”, which assesses the mood-modification component of Facebook addiction, with the residuals of items reflecting on the components of tolerance and withdrawal: item 2 “felt an urge to use Facebook more and more” and item 5 “become restless or troubled if you have been prohibited from using Facebook”. Consistent with our findings, the fit of the Polish and Italian versions of the BFAS was improved among university students and healthy adults by correlating the residuals of several items: item 1 “spent a lot of time thinking about Facebook” and item 2, as well as item 4 “tried to cut down on the use of Facebook” and item 6 “Facebook use has a negative impact on your job/studies” [20, 24, 42]. Accordingly, the authors of those studies suggested that fit improvement in models with correlating item residuals is attributed to a general component of high time and energy investment into the addictive behavior, in addition to the specific core components of addiction [20, 24]. Notably, the loadings of item 3 and its correlation with other items of the French BFAS in a sample of healthy students were exceptionally low. The authors reported a unidimensional structure of the scale. However, they suggested that the BFAS may cover two factors, stressing that non-specificity of item 3 implies that mood modification may not be a mandatory symptom of behavioral addiction in their healthy sample [47].

Unlike these reports, items 2, 3, and 5 had high loadings (.83 to .97) on the corresponding factor in all ED samples, Fig. 1. Investigations of collinearity show that these three items expressed values of variance inflation factor and tolerance within acceptable ranges (< 10 and > 0.1, respectively: Additional file 2). Thus, collinearity does not reflect much impurity of the one-factor structure of the BFAS in our study. In addition, these items had the highest item-total correlations, and their removal from the scale would reduce scale variance and reliability at a degree greater than other variables (Table 5). Thus, we may assume that women with EDs, especially those with AN, use Facebook to escape unpleasant emotions, which are probably the motive for spending more time using Facebook. For the same reason, those patients would experience negative emotions when they quit Facebook. In support of this argument, research denotes that SNSs addiction is derived by a need to escape daily stress, particularly among individuals with moderate-high deficits in emotion regulation [48, 62]. Moreover, experimental induction of negative affect “low-arousal emotions like boredom” followed by exposure to SNSs increased positive affect compared with the use of control websites while experimental induction of positive affect reduced the urge to access SNSs. SNSs had no effect on moderate-intense negative and positive emotions [62]. In addition, a qualitative investigation reports that users’ tendency to stay online more is motivated by the need to complete difficult goals such as achieving satisfaction or reducing fears of missing out. Meanwhile, achieving status or progress as a result of prolonged engagement in online activities (e.g., more reward outcomes in gaming) is associated with diminished mood-modifying effects of the internet [63]. Overall, our findings along with those reported in other studies show that Facebook tolerance in healthy individuals is likely to be motivated by salience, conflict, and relapse components. On the contrary, the need to cope with negative emotions may motivate Facebook tolerance among women with EDs/AN. In line, Facebook use is reported to be similar in patients with irritable bowel syndrome and healthy controls. However, the patient group expressed higher compensatory use while the healthy group demonstrated more Facebook use for socialization [64]. More investigations are needed to define motivational factors of Facebook tolerance in disordered vs healthy groups and the possible effects of these factors on ED pathologies.

This study evaluated invariance of the BFAS across groups of EDs, age, education, and marital status. Because the global fit index *χ*^2^ is sensitive to sample size, absolute fit indices, which are less dependent on sample size, were used to evaluate invariance of the BFAS as reported in the literature [57–59]. Based on the criteria of ΔCFI < .02 and ΔRMSEA < .015, the BFAS was invariant at the configural, metric, scalar, and strict levels across groups of EDs, age, and education. Meanwhile, strict invariance did not hold across groups of marital status. In line, a Peruvian study reported invariance of the BFAS at the configural, metric, scalar, and strict levels across gender groups [43] while an Italian study indicated that one item expressed uniform and small-in-size differential item functioning across gender groups [42]. Only two studies examined invariance of the BFAS across age groups. The Spanish version of the BFAS did not hold scalar invariance across age groups (younger than 20 years and older than 20 years) among Peruvian university students [43]. An Italian study using item response theory revealed non-invariance in 17% of the items of the BFAS across age groups among adolescents and young adults [42]. In addition, strict invariance of the BFAS was not maintained across groups of student academic performance (worsening performance vs non-affected performance) [43]. Over all, the BFAS can be reliably used to compare differences in Facebook addiction between ED groups, especially because scalar invariance was demonstrated by the BFAS in all the models, suggesting low possibility of having serious misinterpretation of true mean differences [58]. In the meantime, one-third the available psychometric measures display partial invariance while strict invariance (demonstrated by the BFAS across marital status) is usually hard to achieve, and thus is overlooked in most validation studies [55, 57, 58].

Criterion validity of the BFAS was proven by SEM and Spearman’s rho correlation with the S-IAT. It is worth mentioning that the BFAS scores did not vary across ED subtypes, but S-IAT scores tended to be lower among women with AN than other EDs (M ± SD = 5.96 ± 6.4 vs 8.7 ± 9.9, t(108.5) = − 1.85, *p* = .067). This result denotes greater risk of addiction to internet activities other than social networking (e.g., online shopping, gaming, etc.) among women with ED subtypes that are characterized by high body mass index/emotional overeating. Although S-IAT strongly correlated with the BFAS in both ED groups (Table 5), its effect among women with other EDs was greater—S-IAT predicted 66% and 88% of the variance in the BFAS in women with AN and other EDs, respectively. In fact, pathological overeating (e.g., BED and BN) represents a phenotype of addictive disorders [65]. EDs characterized by excessive eating, especially when associated with obesity, display multiple alterations in signal transduction, metabolism, and immune response, which increase addictive vulnerability [66, 67]. Although the fit of the unidimensional structure of the BFAS was improved in women with AN by correlating few residuals, this structure exhibited a perfect fit in women with other EDs without correlating any residuals. This may imply that women with EDs characterized by high body mass index typically endorse all addiction components—not necessarily for mood modification.

In relevance to the above-mentioned findings, it is worth mentioning that internet addiction is highly associated with other multiple comorbid addictive behaviors such as food addiction—primarily characterized by binge eating [33], substance abuse, binge drinking, risky sexual behaviors [68], excessive consumption of online pornography, cybersex, and sexual addiction [53, 69, 70]. While binge eating [71, 72] is reported as a genetic risk factor for both EDs and substance abuse, sexual drive and sexual daydreaming are highly reported among women with binge-purging behaviors [73] and hospitalized AN women who managed to reach the mean matched population weight [74]. Accordingly, binge eating and associated genetic risk for substance abuse, along with hypersexuality may increase the possibility that women with EDs would encounter multiple comorbid addictive conditions (substance abuse, binge drinking, social media addiction, internet gaming addiction, sexual addiction, etc.). The risk for the comorbidity of dependencies among this group can be further aggravated by loneliness, being single, and having mental symptoms or a concurrent psychiatric disorder [72, 75]. On the other hand, the co-occurrence of different types of internet addiction and substance abuse in psychiatric patients increases the levels of psychosocial symptoms (primarily depression, obsessive–compulsive symptoms, and interpersonal sensitivity), the chances of meeting criteria for additional mental disorders, and the need for more therapeutic interventions compared with psychiatric patients not meeting criteria for internet addiction [76]. Therefore, it is possible that increased tendency to internet addiction/Facebook addiction among women with other EDs may be associated with more severe and/or prolonged course of EDs. Thus, it is necessary to identify other online addictions (e.g., gaming and cybersex) as well as substance abuse in this patient group for planning of effective interventions.

The internal consistency of the BFAS was excellent in the samples as indicated by high MacDonald’s ω, Cronbach’s α, and corrected-item-total correlations. The latter indicate high discrimination index of the BFAS, i.e., the BFAS can differentiate problematic Facebook users from normal users, which is consistent with previous studies reporting that the BFAS can measure the intensity of Facebook dependence in healthy participants [42, 45].

### Strength, implications, and limitations

This study contributes significantly to the literature by reporting on the psychometric characteristics of the BFAS and its invariance in a clinical sample of women with EDs. The BFAS has a unidimensional structure, which operates evenly across groups of EDs, age, education, and marital status. The results indicate that the BFAS can reliably identify women with EDs who demonstrate high proneness to Facebook addiction. Women with ED subtypes characterized by high body mass index/emotional overeating tend to be more addicted to Facebook and other internet activities than women with AN. Facebook addiction in the latter seems to be a defective way of coping with negative emotions. Thus, Facebook addiction in patients with AN may be mitigated through strategies that target emotion regulation. Assessing Facebook/internet addiction among women with EDs through the BFAS, which demonstrates sound psychometrics, may promote efforts to reduce disorder course through the mitigation of these behavioral comorbidities.

The findings may not be generalized to other clinical populations since they are based on a cross-sectional design involving Spanish patients with EDs from a single healthcare setting. The sample may not be of high statistical power (no sample size calculations were conducted [49]) meanwhile using only 123 ED patients for both CFA and multigroup CFA may be considered a methodological flaw in the current study. Moreover, social desirability and recall biases may be inevitable because data were self-reported. Because the public dataset used in this study does not differentiate subtypes of AN (binge eating/purging type and restricting type), it is not possible to affirm that patients with binge eating/purging AN were not included in the sample of other EDs. Although all participants received and official diagnosis of EDs, it is not clear if the diagnosis was made based on a recognized disease classification system (e.g., DSM-5). We are not able to assume that women with EDs conceptualize Facebook addiction similar to healthy women because of absence of a healthy control group in the study.

## Conclusion

The results show that the BFAS is a unidimensional tool that is mostly invariant across different groups. In addition, the high reliability, discriminant validity, and criterion validity of the BFAS indicate its usefulness for identifying ED patients with Facebook addiction. Investigations of differences in motives of Facebook tolerance in different EDs subtypes are needed in order to guide interventional efforts directed toward ameliorating this form of behavioral addiction.

## Supplementary Information

**Additional file 1. Supplementary Table 1**. Invariance of Model 2 of the Bergen Facebook Addiction Scale (BFAS) across groups of eating disorders.** Supplementary Figure 1**. Structural equation models using the Six-item Internet Addiction Test (S-IAT) to predict the Bergen Facebook Addiction Scale (BFAS) in all the samples.

**Additional file 2.** Additional analysis not included in the manuscript (exploratory factor analysis, BFAS difference across different age groups, multicollinearity of the BFAS.

## Data Availability

The dataset [50] supporting the conclusions of this article is available in Mendeley repository, [https://doi.org/10.1016/j.apnu.2020.07.023], and also the datasets used and/or analyzed during the current study are available from the corresponding author on reasonable request.
