# Supplementary material for: The Bergen Facebook Addiction Scale: its psychometric properties and invariance among women with eating disorders
Source: BMC Womens Health. 2022 Mar 31;22:99. doi: 10.1186/s12905-022-01677-2 (PMC8968775; doi:10.1186/s12905-022-01677-2)
Supplement: Supplementary file 1 — Additional file 1. Supplementary Table 1. Invariance of Model 2 of the Bergen Facebook Addiction Scale (BFAS) across groups of eating disorders. Supplementary Figure 1. Structural equation models using the Six-item Internet Addiction Test (S-IAT) to predict the Bergen Facebook Addiction Scale (BFAS) in all the samples. [file 12905_2022_1677_MOESM1_ESM.docx]

**Appendix A**

**Supplementary Table 1.** Invariance of Model 2 of the Bergen Facebook Addiction Scale (BFAS) across groups of eating disorders

| **Groups** | **Invariance levels** | **χ^2^** | **df** | ***P*** | **Δχ^2^** | **Δdf** | ***p*(Δχ^2^)** | **CFI** | **ΔCFI** | **TLI** | **ΔTLI** | **RMSEA** | **ΔRMSEA** | **SRMR** |
| --- | --- | --- | --- | --- | --- | --- | --- | --- | --- | --- | --- | --- | --- | --- |
| Eating disorders | Configural  Metric  Strong  Strict | 16.690  20.690  25.783  31.374 | 16  21  22  28 | 0.406  0.478  0.261  0.301 | 4.000  5.094  5.591 | 5  1  6 | 0.549  0.024  0.471 | .999  1.000  .996  .996 | -0.001  0.004  0.000 | .999  1.001  .994  .996 | -0.002  0.007  -0.002 | 0.019  0.000  0.038  0.032 | 0.019  -0.038  0.006 | 0.0216  0.0266  0.0581  0.0683 |

| 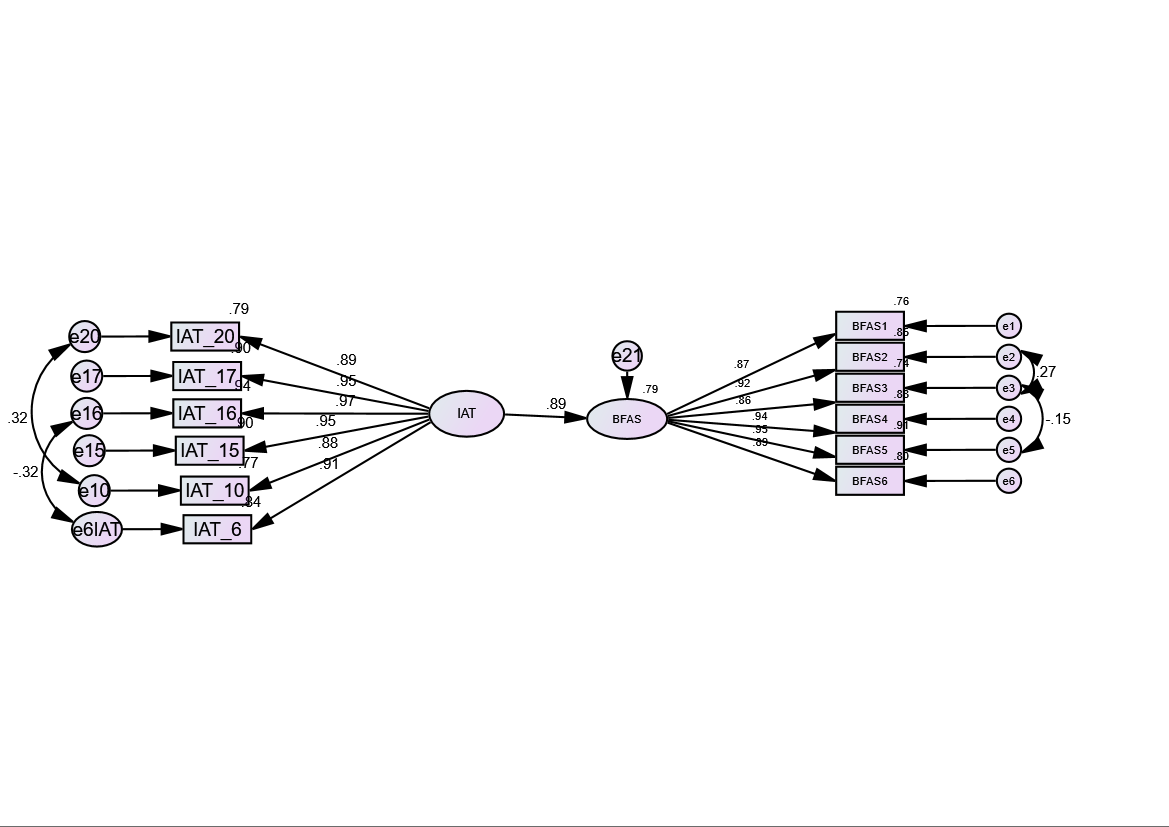  (a) | 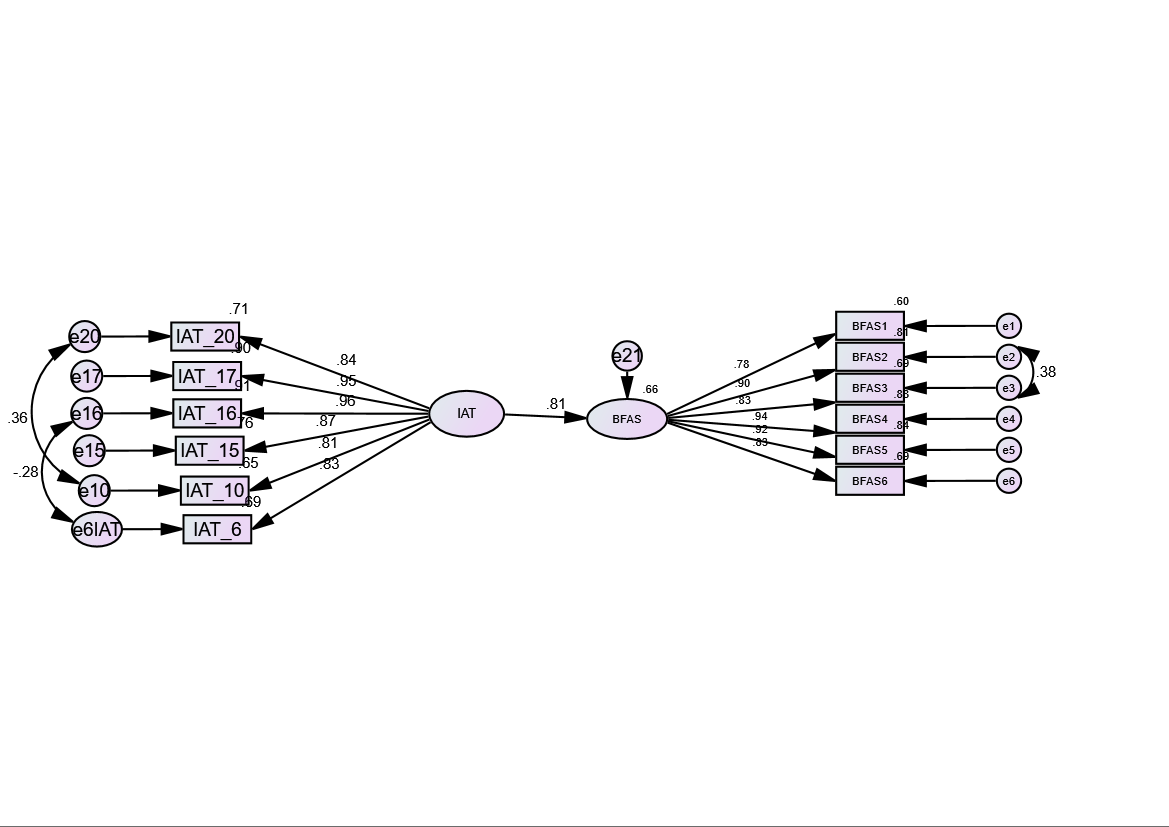  (b) |
| --- | --- |
| 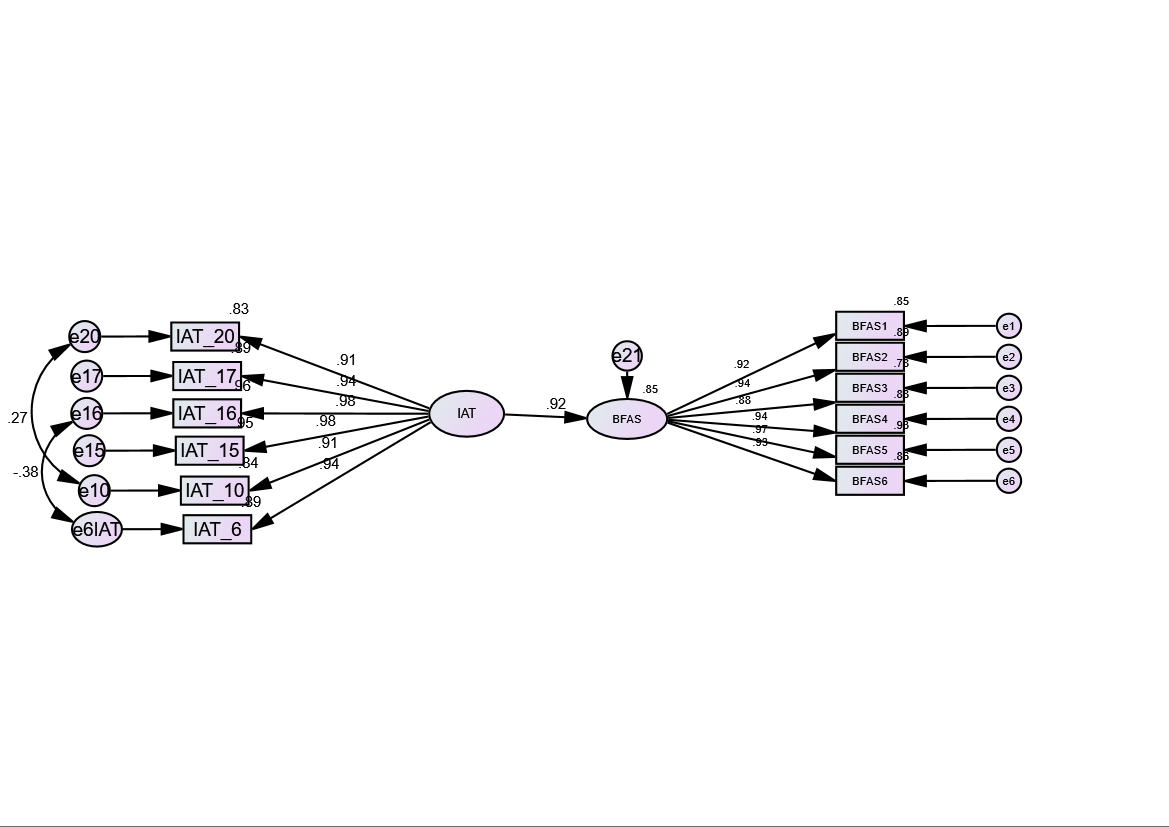  (c) | |

**Supplementary Figure 1.** Structural equation models using the Six-item Internet Addiction Test (S-IAT) to predict the Bergen Facebook Addiction Scale (BFAS) in all the participants (a), women with anorexia nervosa (b), and women with binge eating and bulimia nervosa(c).
